# Association between combinations of nutritional status and quality of life and food purchasing motives among the elderly in South Korea

**DOI:** 10.1186/s12955-020-01434-9

**Published:** 2020-06-16

**Authors:** Doyeon Kim, Hyunjung Lim

**Affiliations:** 1 Research Institute of Medical Nutrition, Kyung Hee University, Seoul, Republic of Korea; 2 Department of Medical Nutrition, Graduate School of East-West Medical Science, Kyung Hee University, Yong-in, Republic of Korea

**Keywords:** Elderly, Food purchasing motives, Nutritional status, Quality of life, South Korea

## Abstract

**Background:**

In the elderly, nutritional status and quality of life (QOL) could potentially affect food purchasing behaviors. We examined the association between combinations of nutritional status and QOL and food purchasing motives among the elderly.

**Methods:**

A total of 143 community-dwelling elderly were recruited in Seoul, South Korea. Nutritional status and QOL were assessed and participants were divided into four groups according to those combinations. Binary logistic regression analysis was used to examine the odds of food purchasing motives according to combinations of nutritional status and QOL.

**Results:**

As a result of comparing the scores (mean ± SD) of the overall important factors for food purchasing, health related factors such as *Nutrition quality* and *Preventive of treatment effect* were the highest score (4.4 ± 0.8), followed by *Price* (4.1 ± 0.9), *Ease of purchase* (3.8 ± 0.9), *Ease of chewing* (3.7 ± 0.9), and *Taste* (3.6 ± 0.9). Participants with a low nutritional status and low QOL had more eating-related problems (77.8%) including chewing difficulty (48.9%) and constipation (17.8%) than those with a high nutritional status and high QOL (*P* < 0.05). Participants who were in high nutritional status and low QOL were more likely to be motivated by *Ease of chewing* (OR: 6.72; 95% CI: 1.44–31.37; *P* < 0.05), while those who were in low nutritional status and high QOL were less motivated by *Taste* (OR: 0.28; 95% CI: 0.08–0.94; *P* < 0.05) compared to those who were in high nutritional status and high QOL.

**Conclusions:**

There were differences in food purchasing motives such as *Ease of chewing* or *Taste *according to combinations of nutritional status and QOL. These data are important in demonstrating differing motives for food choice across nutritional status and QOL, and also provide indications of which care service and food development may be needed in promoting health for the elderly in South Korea.

## Background

The elderly comprise the fastest-growing population group, with WHO projecting a total of 1∙2 billion people over the age of 60 years by 2025 [1]. These trends has important implications of the potential for many people to live a greater number of years in poor health, at risk of malnutrition, with multiple chronic conditions that sometimes translate into functional disability, anorexia, depression, isolation, and loneliness which are dimensions of quality of life (QOL) [2].

QOL focuses on the changes in physical and mental health dimensions that may occur with disease, aging, or decline in functional status [2]. Whereas nutritional status is a description of medically related characteristics that included dietary, anthropometric, biochemical, and clinical indicators of nutritional health [3, 4]. Mini nutritional assessment (MNA) is not only a tool to assess nutritional status, but it is also useful in screening populations to identify frail elderly persons, including factors associated with physical, social, and cognitive domains of the elderly [3]. Thus, it is important to assess nutritional status when evaluating QOL [4]. These dimensions such as functional changes in the elderly may limited food choice due to inability to prepare food or chewing difficulty [4]. Therefore, the important question is to what extent are QOL and nutritional status related to food choice motives or barriers [5].

This study builds upon a growing body of work that focuses on nutritional status and QOL that influence food purchasing motives or barriers related to food choice among the elderly. Several studies have found that attitudes and beliefs underlying food choices among the elderly are rooted in an individual’s physical, social, and cognitive status [5, 6].

Previous study attempted to understand differences in food choices across nutritional status and QOL [4]. Eleni Amarantos (2001) presented age-associated nutritional and QOL changes such as broken bones, edentulous, or missing or false teeth may limit food choices due to inability to prepare food consistency restrictions and decreased income may affect increased food insecurity [4]. These motives or barrier of food choice are at least partly reflected in the existence of QOL and nutritional status. However, there is little published research on what extent the QOL in company with nutritional status may motivate or present barriers to food purchasing among the elderly in South Korea.

This study tested the association between extent of nutritional status and QOL and the various food purchasing motives among the elderly in urban South Korea. We hypothesized that there would be differences in food purchasing motives among combinations of nutritional status and QOL.

## Methods

### Participants

This was a cross-sectional design study in Seoul and Kyunggi area, South Korea. The target population was the users who utilize four local senior welfare center (80%) and one rehabilitation center (20%) in the area, where we contacted by a telephone call. Next, the participants were found through personal contact in the centers from November to December, 2012. Out of a convenience sample of 160 approached, 143 volunteers agreed to participate giving a response rate of 89%. The inclusion criteria were an age of 65 years and older; and having no disability to read and write in Korean and mental disability that precluded completion of the questionnaires; having no acute illness; not being tube-fed; and having no restrictive diet. Participants were administered a questionnaire consisting of items related to socio-demographic, dietary habits, QOL, and MNA that could potentially affect food purchasing, and anthropometric measurements were recorded by trained researchers. We used questionnaire translation version into Korean [7, 8]. Written informed consent was obtained by all participants. The study protocol was approved by the institutional review board (IRB) of Kyung Hee University, Seoul, South Korea (KHSIRB-12-013).

### Measures

To compare anthropometric characteristics, height, weight, body mass index (BMI), waist hip ratio (WHR), mid arm circumference (MAC), and calf circumference (CC) were taken by researchers according to the Centers for Disease Control and Prevention body measurement methods [9]. Measurements were recorded to the nearest 0.1 cm or 0.1 kg. Previous study reported BMI, MAC, and CC predict health status (nutritional status and functional ability) and mortality risk in elderly [10].

For nutritional status, the participants were assessed with MNA, which used for nutritional assessment in geriatric settings [11], and the assessment tool are proposed to be useful in screening malnourished Korean elderly patients [8, 12–14]. The MNA gives a maximum of 30 points: a score less than 17.0 points is considered to indicate malnutrition; 17.0 to 23.5 indicates a risk for malnutrition; and 24.0 points or more indicates a good nutritional status. In this study, MNA is divided by two category: high and low nutritional status which indicated ‘well nourished (MNA ≥ 24)’ and ‘at risk of malnutrition or malnutrition (MNA ≤ 23.5)’, respectively. To assess QOL, and we used the Korean version of SF-36 which was demonstrated acceptable validity and reliability among healthy elderly people and elderly patients in South Korea [7]. All questions are scored on a scale from 0 to 100, with 100 representing the highest level of functioning possible. Aggregate scores are compiled as a percentage of the total points possible and averaged together, for a final score within each of the 8 dimensions measured (e.g., pain, physical function, etc.). And then, add all final score of 8 categories [7, 15]. In our study, QOL was categorized by percentage: high and low QOL, which indicated ‘better QOL (≥ 50%)’ and ‘worse QOL (< 50%)’, respectively. Lastly, we defined four nutritional status-QOL groups, namely, high nutritional status and high QOL (best health), high nutritional status and low QOL, low nutritional status and high QOL, and low nutritional status and low QOL (worst health). Information recorded in the factors influencing food purchasing decisions included the how influential the factors [6, 16, 17] were on food purchasing behaviors on a scale from one (not at all important) to five (very important). The following food purchasing factors were assessed: taste, ease of chewing, price, ease of opening the package, preventive or treatment effect on disease, nutrition quality, length of cooking time, and ease to purchase.

For sample size, we expected to have 95% a priori power based on the multiple regression ρ2 of 0.16 [18], seven predictors, and α = 0.05. The sample size was calculated using G*power software, version 3.0 (University kiel, Germany).

### Statistical analysis

All statistical analyses were performed using the Statistical Package for Social Science (SPSS, version 21.0, IBM Corporation, Chicago, Illinois, USA). Descriptive statistics (mean, SD and proportions) were computed for socio-demographic, anthropometric data, dietary habit, and food purchasing motives in the participants according to nutritional status, QOL and nutritional status & QOL combination groups. Group comparisons were made using Student’ *t* test for continuous data and χ^2^ test for catogorical data. Lastly, we performed a binary logistic regression analyses between nutritional status & QOL combination groups and food purchasing motive variables using participants who were in high nutritional status & high QOL as reference group, adjusted for age, sex, marital status, education, income, and number of chronic diseases. For the binary logistic regression analyses, response categories for motivations were collapsed into not important (not at all important, little important, and neutral) versus important (moderately important and very important). Significance was set at* P*<0.05.

## Results

### Socio-demographic characteristics

The socio-demographic characteristics of the participants across nutritional status, QOL and nutritional status & QOL are shown in Table 1. The mean age of the subject was 75.1 ± 5.5 y. Participants with low QOL were more females, older, had a lower education and income, more chronic diseases and medication use, and were less likely to exercise regularly than participants with high QOL. Similarly, participants with low nutritional status had a higher proportion of older and a lower education (*p* < 0.05).

**Table 1:** Socio-demographic characteristics according to nutritional status, QOL and MNA & QOL of the elderly in South Korea (*N* = 143)

	Nutritional status^**a**^			QOL^**b**^			Nutritional status & QOL^**c**^		
	High (*N* = 63)	Low (*N* = 68)	*P*^d^	High (*N* = 71)	Low (*N* = 70)	*P*	Best (*N* = 41)	Worst (*N* = 45)	*P*
**Female**^**e**^	66.7	66.2	0.953	54.9	74.3	**0.016**	58.5	71.1	0.177
**Age**^**f**^	74.4 ± 4.7	75.9 ± 6.2	0.105	73.6 ± 5.5	76.5 ± 5.2	**0.001**	73.4 ± 5.1	77.0 ± 5.9	**0.003**
< 80 years	88.9	73.5	**0.025**	87.3	75.7	0.076	90.2	68.9	0.072
≥ 80 years	11.1	26.5		12.7	24.3		9.8	31.1	
**Disease (multiple response)**	82.0	85.3	0.609	74.3	92.8	**0.003**	72.5	88.9	**0.027**
Diabetes	21.3	20.6	0.920	17.1	24.6	0.277	12.5	17.8	0.107
Hypertension	57.4	47.1	0.242	51.4	53.6	0.796	52.5	46.7	0.478
Hyperlipidemia	13.1	17.6	0.478	8.6	21.7	**0.030**	7.5	20.0	0.277
Gastrointestinal disease	4.9	11.8	0.164	10.0	5.8	0.359	5.0	6.7	0.095
Stroke	1.6	16.2	**0.005**	2.9	15.9	**0.008**	2.5	22.2	**0.003**
Bone joint disease	16.4	26.5	0.166	7.1	33.3	**0.000**	2.5	31.1	**0.001**
**Education level**									
None	1.6	11.8	**0.027**	4.2	10.0	**0.000**	2.4	13.3	**0.005**
Elementary school	25.4	32.4		15.5	40.0		14.6	37.8	
Middle school	14.3	20.6		14.1	20.0		12.2	22.2	
High school	27.0	20.6		26.8	21.4		26.8	17.8	
≥ College	31.7	14.7		39.4	8.6		43.9	8.9	
**Income (US$)**									
< 1000	62.9	75.0	0.357	55.7	82.9	**0.007**	55.0	84.4	0.137
1000 - 2000	25.8	17.6		30.0	11.4		30.0	8.9	
2000 - 3000	1.6	2.9		4.3	1.4		2.5	2.2	
≥ 3000	9.7	4.4		10.0	4.3		12.5	4.4	
**Marital status**									
Living with partner	47.6	38.8	0.208	52.1	37.7	0.053	51.2	34.1	0.272
Divorced or separated	7.9	6.0		9.9	2.9		12.2	4.5	
Widowed	42.9	49.3		35.2	55.1		34.1	54.5	
Single and never married	1.6	0.0		1.4	0.0		2.4	0.0	
**Medication**	77.4	85.3	0.248	74.6	88.4	**0.036**	68.3	86.7	**0.042**
**Exercise regularly**	84.1	77.9	0.368	87.3	74.3	**0.049**	92.7	75.6	0.076
**Alcohol drinkers**	14.3	5.9	0.108	18.3	5.7	**0.022**	14.6	0.0	0.051
**Smoking**									
Current	3.2	5.9	0.579	2.8	5.7	0.612	2.4	6.7	0.754
Past	25.4	29.4		26.8	24.3		31.7	31.1	
Never	71.4	63.2		70.4	68.6		65.9	60.0	

Abbreviations: *MNA* mini nutritional assessment, *QOL* quality of life

^a^MNA is divided by two categries: high (≥ 24) and low nutritional status (≤ 23.5)

^b^QOL, assessed score using the SF-36, was categoried by percentage: high (≥ 50%) and low (< 50%) QOL

^c^Nutritional status & QOL were grouped by combinations of each category of MNA and QOL. Best, high nutritional status and high QOL; worst, low nutritional status and low QOL

^d^*P*-values for differences between groups using Chi-square test for proportions and Student's t test for mean. Values in boldface are significant (*p* < 0.05)

^e^Values are expressed as percentages

^f^Values are expressed as means ± SD

### Anthropometric characteristics

Comparison of anthropometric characteristics across nutritional status, QOL and nutritional status & QOL are shown in Table 2. Participants with low nutritional status had less BMI, MAC and CC than those with high nutritional status. In addition, participants with low nutritional status & low QOL and low QOL had less CC compared to high nutritional status & high QOL and high QOL group, respectively (*p* < 0.05).

**Table 2:** Anthropometric characteristics according to nutritional status, QOL, and nutritional status & QOL of the elderly in South Korea (*N* = 143)

	Nutritional status^**a**^			QOL^**b**^			Nutritional status & QOL^**c**^		
	High (*N* = 63)	Low (*N* = 68)	*P*^d^	High (*N* = 71)	Low (*N* = 70)	*P*	Best (*N* = 41)	Worst (*N* = 45)	*P*
Height (cm)^e^	155.9 ± 6.6	155.5 ± 8.1	0.768	157.3 ± 7.4	154.4 ± 7.3	**0.018**	156.7 ± 7.0	154.4 ± 8.0	0.161
Weight (cm)	62.6 ± 8.3	56.8 ± 8.2	**0.000**	60.1 ± 8.5	59.3 ± 9.2	0.590	61.5 ± 8.2	56.5 ± 8.3	**0.007**
BMI (kg/m^2^)	25.8 ± 3.5	23.5 ± 3.1	**0.000**	24.3 ± 3.2	24.9 ± 3.7	0.277	25.0 ± 3.1	23.7 ± 3.2	0.055
Underweight (< 18.5)^f^	0.0	5.9	**0.022**	1.4	4.3	0.380	0.0	6.7	0.082
Normal weight (18.5–22.9)	22.2	38.2		36.6	25.7		29.3	33.3	
Overweight (23.0–24.9)	25.4	23.5		23.9	22.9		26.8	24.4	
Obese (≥25.0)	52.4	32.4		38.0	47.1		43.9	35.6	
WHR	0.88 ± 0.05	0.89 ± 0.07	0.204	0.88 ± 0.05	0.89 ± 0.07	0.139	0.88 ± 0.04	0.90 ± 0.07	0.100
MAC (cm)	28.1 ± 2.6	26.4 ± 2.2	**0.000**	26.8 ± 2.2	27.6 ± 2.9	0.059	27.3 ± 1.9	26.5 ± 2.2	0.089
CC (cm)	35.0 ± 2.8	32.0 ± 3.1	**0.000**	34.0 ± 3.0	32.8 ± 3.4	**0.020**	34.8 ± 3.0	31.5 ± 3.3	**0.000**

Abbreviations: *MNA* mini nutritional assessment, *QOL* quality of life, *BMI* body mass index, *WHR* waist hip ratio, *MAC* Mid-arm circumference, *CC* Calf Circumference

^a^MNA is divided by two categries: high (≥ 24) and low nutritional status (≤ 23.5)

^b^QOL, assessed score using the SF-36, was categoried by percentage: high (≥ 50%) and low (< 50%) QOL

^c^Nutritional status and QOL were grouped by combinations of each category of MNA and QOL. Best, high nutritional status and high QOL; worst, low nutritional status and low QOL

^d^*P*-values for differences between groups using Chi-square test for proportions and Student's t test for mean. Values in boldface are significant (*p* < 0.05)

^e^Values are expressed as means ± SD

^f^Values are expressed as percentages

### Dietary habit

Results related to dietary behaviors across nutritional status, QOL and nutritional status & QOL are shown in Table 3. Anorexia, as a reason for meal skipping, and eating-related problems included chewing difficulty, indigestion, or constipation were more likely to be in low nutritional status & low QOL compared to high nutritional status & high QOL. Participants with low QOL had more frequent snacking compared to high QOL group. Lastly, they reported eating less than usual compared to participants with high nutritional status & high QOL and high QOL (*p* < 0.05).

**Table 3:** Dietary habits according to nutritional status, QOL and nutritional status & QOL of the elderly in South Korea (*N* = 143)

	Nutritional status^**a**^			QOL^**b**^			Nutritional status & QOL^**c**^		
	High (*N* = 63)	Low (*N* = 68)	*P*^d^	High (*N* = 71)	Low (*N* = 70)	*P*	Best (*N* = 41)	Worst (*N* = 45)	*P*
**Meal frequency**^**e**^			0.302			0.189			0.214
3 times a day	92.1	83.8		84.5	92.9		87.8	88.9	
2 times a day	6.3	14.7		12.7	7.1		9.8	11.1	
1 times a day	1.6	1.5		2.8	0.0		2.4	0.0	
**Reason for meal skipping** (multiple response)									
Sleep lately	0.0	3.3	0.497	1.7	1.6	1.000	0.0	2.3	0.436
Anorexia	0.0	13.3	**0.007**	5.1	7.8	0.719	0.0	11.6	**0.038**
Indigestion	0.0	8.3	0.059	1.7	6.3	0.367	0.0	9.3	0.168
Difficulty of food preparation	0.0	6.7	0.120	3.4	3.1	1.000	0.0	4.7	0.136
**Eating-related problem** (multiple response)	28.6	66.2	**0.000**	26.8	65.7	**0.000**	19.5	77.8	**0.000**
Swallowing problems	0.0	3.2	0.498	1.6	1.5	1.000	0.0	2.2	0.445
Chewing difficulty	17.9	42.9	**0.003**	18.0	41.2	**0.004**	13.9	48.9	**0.007**
Anorexia	3.6	7.9	0.445	4.9	7.4	0.721	2.8	6.7	0.661
Indigestion	5.4	17.5	**0.041**	9.8	16.2	0.288	2.8	15.6	0.146
Nausea / vomiting	0.0	1.6	1.000	0.0	1.5	1.000	0.0	2.2	0.646
Constipation	3.6	12.7	0.101	0.0	14.7	**0.002**	0.0	17.8	**0.017**
**Snacks frequency**									
3 times a day	7.9	10.3	0.387	5.6	11.4	**0.025**	2.4	8.9	0.081
2 times a day	20.6	30.9		22.5	28.6		19.5	31.1	
1 times a day	44.4	33.8		36.6	37.1		48.8	40.0	
Every other day	3.2	7.4		1.4	8.6		2.4	11.1	
Almost never	23.8	17.6		33.8	14.3		26.8	8.9	
**Frequency of dining-out**									
1 times a day	21.0	19.1	0.075	18.3	18.8	**0.010**	22.0	20.0	0.067
1–6 times a week	22.6	22.1		29.6	13.0		29.3	15.6	
1–3 times a month	33.9	17.6		31.0	23.2		31.7	15.6	
Almost never (less than once a month)	22.6	41.2		21.1	44.9		17.1	48.9	
**Change food amount** (recently)									
No change	88.9	75.0	0.109	88.7	71.4	**0.004**	95.1	68.9	**0.015**
More-than-usual-intake	1.6	5.9		5.6	2.9		2.4	4.4	
Less-than-usual-intake	9.5	19.1		5.6	25.7		2.4	26.7	

Abbreviations: *MNA* mini nutritional assessment, *QOL* quality of life

^a^MNA is divided by two categries: high (≥ 24) and low nutritional status (≤ 23.5)

^b^QOL, assessed score using the SF-36, was categoried by percentage: high (≥ 50%) and low (< 50%) QOL

^c^Nutritional status and QOL were grouped by combinations of each category of MNA and QOL. Best, high nutritional status with high QOL; worst, low nutritional status with low QOL

^d^*P*-values for differences between groups using Chi-square test for proportions. Values in boldface are significant (*p* < 0.05)

^e^Values are expressed as percentages

### Food purchasing motives

**Fig. 1:**
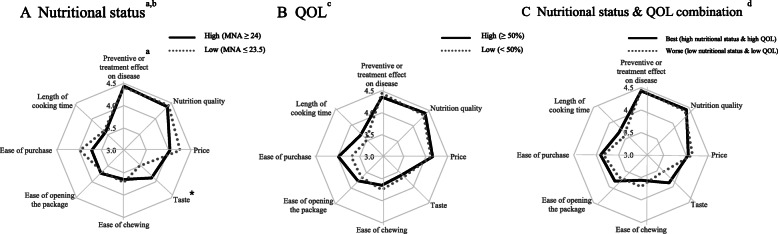
Comparison of food purchasing motives by nutritional status, QOL, and nutritional status & QOL combinations in the elderly in South Korea. **a**: High nutritional status, solid line; low nutritional status, dotted line. **b**: high QOL (≥50%); low QOL (< 50%), dotted line. **c**: high nutritional status & high QOL, solid line; low nutritional status & low QOL, dotted line. ^a^Food purchasing motives were selected by the ranking among items. Motivations were rated by 1 (‘not at all important’), 2 (‘a little important’), 3 (‘neutral’), 4 (‘moderately important’), and 5 (‘very important’) scale. ^b^ MNA was categoried by score: high nutritional status (MNA ≥ 24) and low nutritional status (MNA < 24). ^c^ QOL, assessed score using the SF-36, was categoried by percentage: high (≥ 50%) and low (< 50%).^d^Nutritional status and QOL was grouped by combinations of each category of MNA and QOL: high nutritional status & high QOL, low nutritional status & low QOL. *P < 0.05, between-group difference was significant

The responses to the question “What is the most important factor in your decisions about the foods you purchase?” were shown in Fig. 1. A comparison of overall important factor for food purchasing, health related factor such as *Nutrition quality* (4.4 ± 0.7) and *Preventive of treatment effect* (4.4 ± 0.8) were the highest score, followed by *Price* (4.1 ± 0.9), *Ease of purchase* (3.8 ± 0.9), *Ease of chewing* (3.7 ± 1.0), and *Taste* (3.6 ± 1.0). *Taste* was less motivated factor in the low nutritional status compared to high nutritional (*p* < 0.05).

### Food purchasing motives across nutritional status and QOL

The results of the binary logistic regressions of motivations for food purchasing motives according to nutritional status & QOL aged 65 years and older are reported in Table 4. Participants who were in the high nutritional status & low QOL were more likely to be motivated by *Ease of chewing* (OR: 6.72; 95% CI: 1.44–31.37; *P* < 0.05) compared to high nutritional status & high QOL adjusted for age, sex, marital status, education, income, and number of chronic diseases (*p* < 0.05). Participants who were in low nutritional status & high QOL were less likely to be motivated in *Taste* (OR: 0.28; 95% CI: 0.08–0.94; *P* < 0.05) compared to high nutritional status & high QOL adjusted for covariates (*p* < 0.05).

**Table 4:** Logistic regressions of food purchasing motives according to nutritional status & QOL of the elderly in South Korea (*N* = 143)^a^

	Unadjusted^**b**^		Adjusted^**c**^	
	OR (95% CI)	*P*^d,e^	OR (95% CI)	*P*
**Taste**				
high nutritional status & high QOL	1.000		1.000	
high nutritional status & low QOL	0.887 (0.289–2.721)	0.834	0.830 (0.207–3.326)	0.793
low nutritional status & high QOL	**0.318 (0.110–0.922)**	**0.035**	**0.279 (0.083–0.939)**	**0.039**
low nutritional status & low QOL	0.544 (0.222–1.338)	0.185	0.589 (0.192–1.804)	0.354
**Ease of chewing**				
high nutritional status & high QOL	1.000		1.000	
high nutritional status & low QOL	**3.570 (1.108–11.504)**	**0.033**	**6.715 (1.438–31.365)**	**0.015**
low nutritional status & high QOL	1.633 (0.579–4.609)	0.354	1.837 (0.579–5.832)	0.302
low nutritional status & low QOL	1.838 (0.772–4.374)	0.169	1.716 (0.604–4.880)	0.311
**Price**				
high nutritional status & high QOL	1.000		1.000	
high nutritional status & low QOL	1.011 (0.315–3.249)	0.985	0.977 (0.219–4.364)	0.976
low nutritional status & high QOL	2.529 (0.625–10.233)	0.193	3.260 (0.722–14.716)	0.124
low nutritional status & low QOL	1.433 (0.522–3.937)	0.485	2.107 (0.572–7.759)	0.263
**Ease of opening the package**				
high nutritional status & high QOL	1.000		1.000	
high nutritional status & low QOL	0.538 (0.187–1.552)	0.252	0.279 (0.071–1.099)	0.068
low nutritional status & high QOL	1.010 (0.344–2.962)	0.986	0.698 (0.209–2.334)	0.560
low nutritional status & low QOL	1.005 (0.407–2.480)	0.991	0.536 (0.169–1.700)	0.289
**Preventive or treatment effect on disease**				
high nutritional status & high QOL	1.000		1.000	
high nutritional status & low QOL	2.333 (0.244–22.281)	0.462	2.419 (0.207–28.308)	0.481
low nutritional status & high QOL	0.528 (0.119–2.349)	0.402	0.693 (0.140–3.430)	0.653
low nutritional status & low QOL	0.685 (0.178–2.632)	0.582	1.333 (0.267–6.649)	0.726
**Nutrition quality**				
high nutritional status & high QOL	1.000		1.000	
high nutritional status & low QOL	2.857 (0.311–26.207)	0.353	2.590 (0.231–29.046)	0.440
low nutritional status & high QOL	1.500 (0.267–8.434)	0.645	3.520 (0.440–28.139)	0.235
low nutritional status & low QOL	0.905 (0.253–3.230)	0.877	2.660 (0.472–15.005)	0.268
**Length of cooking time**				
high nutritional status & high QOL	1.000		1.000	
high nutritional status & low QOL	0.512 (0.178–1.471)	0.214	0.585 (0.161–2.127)	0.415
low nutritional status & high QOL	1.386 (0.479–4.011)	0.547	1.768 (0.542–5.770)	0.345
low nutritional status & low QOL	1.027 (0.429–2.455)	0.953	1.499 (0.512–4.387)	0.460
**Ease to purchase**				
high nutritional status & high QOL	1.000		1.000	
high nutritional status & low QOL	0.339 (0.112–1.026)	0.055	0.382 (0.100–1.455)	0.159
low nutritional status & high QOL	1.966 (0.551–7.005)	0.297	2.259 (0.602–8.471)	0.227
low nutritional status & low QOL	0.772 (0.308–1.938)	0.582	0.951 (0.324–2.790)	0.927

Abbreviations: *MNA* mini nutritional assessment, *QOL* quality of life, *OR* odds ratio, *CI* confidence interval, *ref* reference

^a^Nutritional status and QOL are grouped by combinations of each category of nutritional status and QOL

^b^Unadjusted result of logistic regression analysis

^c^Adjusted for age, sex, marital status, education, income, and number of chronic diseases

^d^Trend analysis for the null hypothesis that OR = 1.0 (ref =  high nutritional status & high QOL)

^e^Values in boldface are significant at *p* < 0.05

## Discussion

This present study investigated the association between the extent of nutritional status and QOL combination and the various food purchasing motives among the elderly in urban South Korea. In overall participants, the results observed that *Preventive or treatment effect* and *Nutrition quality* were rated as the most important among the food purchasing motives. The group with high nutritional status & low QOL was more likely to be associated with considering *Ease of chewing* as food purchasing motives, whereas the group with low nutritional status & high QOL was less likely to be associated with considering *Taste* as food purchasing motives among the urban elderly.

Based on the MNA criteria, 48.1% of participants were ‘well nourished’ and 51.9% of participants were ‘at risk of malnutrition’ or ‘malnutrition’ status. In previous studies, malnutrition was considered to be one of the most relevant conditions that negatively affected the health status (alteration of the immune system, muscle loss, function of impairment) of the elderly in various care settings [19, 20]. In addition, QOL provides a validated approach for expanding the definition of health to include other domains of mental and social well-being, especially physical functioning, as assessed with the activities of daily living (ADL) scores [2]. Impaired mobility, inability to feed oneself, or chewing difficulty might change dietary habits and further induce malnutrition [2]. Also, lower QOL was associated more with the frail compared to the non-frail [21], as well as MNA, which is a useful screening tool to identify frail elderly [3]. Therefore, participants with low nutritional status & low QOL might be more vulnerable to physical and mental health, social functioning, and emotional well-being in the elderly [22]. Our previous findings indicate that nutritional status, diet quality and physical performance were independently and significantly inversely associated with IL-6 or TNF-α in frail elderly which suggested that nutritional status is associated with overall quality of life including physical status [23]. Therefore, dietary behavior is important to maintain individual health.

Food purchasing behavior is related to ‘food choice’ which is a complex process including cultural, socio-, and psychological factors that varied within individuals and had different strengths among the various groups of people and for different foods [24]. Items important in food choice involved taste, health, convenience, price, ease of chewing, and traditional beliefs [24]. Many studies have represented that chewing difficulty is correlated to loss of teeth, and that food choice in the elderly is often of poor nutritional value. However, some researcher have presented that there are many factors that influence food choice, and improving prosthesis quality is not necessarily going to result in alteration of food choice behavior, but rather, educational level is more strongly influential [25]. The fact represents that intervention including nutritional education to help the elderly alter their food choice to include foods with a high nutritional value [26] is more useful. In high nutritional status & low QOL more considered *Ease of chewing* as food purchasing motives in the elderly. In dietary habit, there are less than usual intake and more eating-related problem such as chewing difficulty in low QOL group, and it implied that high nutritional status and low QOL group have the most barriers in choosing food in oral health, and maybe have potential risk for dietary inadequacy. In addition, as people age, taste sensation tends to diminish, and this may affect appetite [25]. In our result, low nutritional status & high QOL group less considered *Taste* as a food purchasing motive among the elderly. In dietary habits according to nutritional status, low nutritional group answered ‘anorexia’ is the reason for meal skipping, and probably the fact influenced low nutritional status & high QOL group. Further research is required into methods of food preparation which may increase appetite for the elderly group. Besides, other food purchasing motives such as *Ease of opening packages, Length of cooking time,* and *Ease of purchase*, were indicators of convenience not significantly associated with nutritional status & QOL categories [27].

The current study has a number of strength. First, most previous studies were only conducted according to nutritional status or QOL, but we investigated the combinations of nutritional status and QOL categories. Second, this work may be a first step toward the development of evidence based behavioral nutritional intervention for the elderly suffering from dietary inadequacies (e.g., anorexia or chewing difficulty) who were in poor quality of life or malnutrition. Efforts to change dietary behaviors, especially community-based interventions involving self-management approaches, must carefully take into account individual food purchasing motives in order to be successful.

This study was limited by its reliance on measures of self-reporting. Additionally, while this work focused on community-based elderly, we might expect that findings from this study extend to other groups as well. Further studies are necessary to evaluate whether these finding hold up for other geographic areas and ethnic groups.

## Conclusions

In summarty, we found that physical well-being related motives, *Preventive or treatment effect* and *Nutrition quality* were rated as the most important among the food purchasing motives among the urban elderly. Second, the group with high nutritional status & low QOL was more likely to be associated with considering *Ease of chewing* as food purchasing motives, whereas the group with low nutritional status & high QOL was less likely to be associated with considering *Taste* as food purchasing motives among the elderly in urban South Korea. This result suggests that a customized approach according to QOL and nutritional status be taken in medical nutrition therapy for the elderly for ‘healthier’ food purchasing behaviors.

## Data Availability

The datasets used and/or analyzed during the current study are available from the corresponding author on reasonable request.

## Abbreviations

QOL: Quality of life
ADL: Activities of daily living
MNA: Mini nutritional assessment
IRB: Institutional review board
BMI: Body mass index
WHR: Waist hip ratio
MAC: Mid arm circumference
CC: Calf circumference
PF: Physical functioning
RP: Role limitation due to physical health
BP: Body pain
GH: General health perceptions
VT: Vitality
SF: Social functioning
RE: Role limitations as a result of emotional problems
MH: General mental health
